# Surgical Outcomes of Newly Trained ShangRing Circumcision Providers

**DOI:** 10.1097/QAI.0000000000000750

**Published:** 2016-05-24

**Authors:** Quentin D. Awori, Richard K. Lee, Philip S. Li, Robert Zulu, Kawango Agot, Stephanie Combes, Raymond O. Simba, Catherine Hart, Jaim Jou Lai, Zude Zyambo, Marc Goldstein, Paul J. Feldblum, Mark A. Barone

**Affiliations:** * ShangRing Research, EngenderHealth, Nairobi, Kenya;; † Center for Male Reproductive Medicine and Surgery, Department of Urology, Weill Cornell Medical College, New York Presbyterian Hospital, New York, NY;; ‡ Department of Urology, University Teaching Hospital, Lusaka, Zambia;; § VMMC programme, Impact Research & Development Organization, Kisumu, Kenya;; ‖ Clinical Sciences Unit, FHI 360, Durham, NC;; ¶ Surgical Department, Homa Bay District Hospital, Homa Bay, Kenya;; # ZPCT, FHI 360, Lusaka, Zambia; and; ** Clinical Research, EngenderHealth, New York, NY.

**Keywords:** male circumcision, ShangRing, circumcision device, training, outcomes, skill

## Abstract

**Background:**

Devices can potentially accelerate scale-up of voluntary medical male circumcision in sub-Saharan Africa. Studies have demonstrated advantages of the ShangRing device over conventional circumcision. With the need to train providers rapidly for scale-up, concerns arise about the transferability of techniques and the expertise of new trainees.

**Methods:**

We compared outcomes of ShangRing circumcisions conducted in Kenya by experienced providers (experience with more than 100 ShangRing circumcisions) and newly trained providers (trained in Kenya by the experienced providers before the study began). During training, trainees performed at least 7 ShangRing circumcisions and 3 removals. Newly trained providers received intermittent clinical mentoring initially during the study but otherwise conducted circumcisions on their own.

**Results:**

Four hundred six and 115 ShangRing procedures were performed by the new trainees and the experienced providers, respectively. The mean duration of circumcisions was 6.2 minutes for both trained and experienced provider groups (*P* = 0.45), whereas the mean pain score (on an 11-point scale) was 2.5 and 3.2, respectively (*P* = 0.65). There was no difference in the proportion of participants healed by the day 42 visit (*P* = 0.13) nor in the incidence of moderate and severe adverse events observed (*P* = 0.16). Participants in both groups were equally satisfied with final wound cosmesis.

**Discussion:**

Results demonstrate that the ShangRing circumcision technique is easy to learn and master. Newly trained providers can safely conduct ShangRing circumcisions in routine service settings. The ShangRing can facilitate rapid rollout of voluntary medical male circumcision for HIV prevention in sub-Saharan Africa.

## INTRODUCTION

Voluntary medical male circumcision (VMMC) is one of the main strategies used to prevent the transmission of HIV. In 3 randomized control trials (RCTs) conducted in South Africa, Kenya, and Uganda, male circumcision (MC) was found to reduce the chances of female-to-male transmission of HIV through penile-vaginal intercourse by up to 60%.^1–3^ Further studies have found this long-term effect of protection to be sustained at up to 73% up to 5 years after circumcision.^4–6^

Based on the original findings of the 3 RCTs and recommendations from the World Health Organization (WHO) and the Joint United Nations Programme on HIV/AIDS (UNAIDS), 14 sub-Saharan African countries have been implementing VMMC in their national HIV control programs.^7,8^ The aim of the joint WHO/UNAIDS action framework is to have a VMMC prevalence of at least 80% among 15- to 49-year-old males and have established a sustainable national programme that provides VMMC services to all infants up to 2 months old and at least 80% of male adolescents. This is to be achieved by 2016 in countries with generalized HIV epidemics and low prevalence of MC.^9^ A rapid scale-up of circumcision was hence initiated.^10^ Despite considerable progress, it is unlikely that this goal will be met within the timeline.^11^

Strategies to rapidly scale up the implementation of VMMC have included the use of device-assisted MC, which has the potential to reduce the circumcision time and may create some demand among those who have avoided conventional circumcision.^11^ An ideal circumcision device should be easy to learn how to use, safe and acceptable to the client, and should provide reproducible quality results.^12^ The ShangRing is one such device that has been found to be safe, acceptable, and effective. It has been studied in China (its country of origin), Kenya, Zambia, and Uganda in adult men and adolescents.^13–15^

Rapid scale-up of VMMC using devices would require the training of new circumcision providers and retraining of those already providing VMMC. In sub-Saharan Africa, a human resource of approximately 2282 male circumcision providers (MCPs) per 10,000 men and 513 MCPs per 10,000 men would be needed in the catch-up and sustainability phases, respectively.^16^ With the acknowledged need to train more providers, issues concerning transferability of skills arise. In an analysis of adverse event (AE) risk factors following VMMC, Frajzyngier et al^17^ found that clients circumcised by nurses were more likely to experience an AE compared with those circumcised by clinical officers, although the increased AE rate was still within the published acceptable ranges and that the number of years as a professional had no bearing on the likelihood of the client experiencing an AE.

The objective of this analysis was to compare surgical outcomes for the ShangRing circumcision procedure between experienced and newly trained MCPs.

## METHODS

Data for this analysis were drawn from a prospective observational study of adult MC using the ShangRing performed at 6 sites in Kenya and 3 sites in Zambia. The primary objective of the study was to estimate the rates of AEs, particularly those that are rare or unexpected, after routine service delivery of a ShangRing circumcision. Details of the study design, methods, and main results have been previously published.^13^

Before the study, inexperienced providers were trained to perform the ShangRing circumcision and removal technique in each country by providers who were already experienced in the ShangRing technique. In Kenya, 6 experienced ShangRing providers trained a group of 13 MC providers who had varied experience with conventional circumcision techniques. A ShangRing curriculum was used to conduct the training; it included both didactic lectures (1.5 days) and a practical component (3.5 days). During the practical sessions, each trainee performed at least 7 ShangRing circumcisions and 3 removals, being assisted by either a trainer or a fellow trainee, before being deemed as having acquired the skill.

In Kenya, 10 newly trained ShangRing providers were distributed in pairs to 5 peripheral sites. The experienced providers were stationed at the main study site. During the first few weeks, the newly trained ShangRing providers received on-site supportive supervision (from 3 of the experienced providers at the main site) during which any difficulties they had in conducting the procedures were addressed. Thereafter, the newly trained MCPs performed the circumcisions without on-site assistance or supervision. They were, however, able to reach the experienced ShangRing providers when needed for prompt advice through phone.

In Zambia, each of the experienced MCPs partnered with the newly trained MCPs to form 3 teams, each of which conducted ShangRing circumcisions at 1 of the study sites. Since the newly trained and experienced providers worked together in the Zambia sites, data from this arm were excluded from this analysis.

Both HIV-positive and HIV-negative clients were enrolled into the study. After obtaining informed consent, recruited participants underwent clinical examination to confirm eligibility. All clients were given 1 gram of paracetamol approximately 30 minutes before the circumcision procedure so that its onset of action would commence after the local anesthesia had worn off. Local anesthesia was administered through dorsal penile nerve block and circumferentially around the penile shaft (ring block) using 1% lidocaine. Clients underwent ShangRing circumcision as previously described.^18^ Two providers were involved in each circumcision procedure with one taking the lead while the other assisted.

After the circumcision, participants were to return to the clinic 7 days later, for ring removal. They were then scheduled for a single visit 42 days after the circumcision to assess healing. All clients were asked to contact their respective study sites if they experienced any untoward event such as bleeding from the surgical site, pain or swelling that got increasingly worse, a foul smell, lower abdominal pain, difficulty passing urine, or developed a fever.

### Outcomes

ShangRing circumcision and removal times, pain scores of participants, time to complete healing, interview responses concerning participant satisfaction, and AEs' rates were assessed to compare the outcomes of the newly trained and experienced MCPs.

Procedure times were measured from the placement of the inner ring until after the foreskin was removed and slits created on the remnant skin; it did not include the time taken for the injected anesthesia to take effect. Removal times were measured from the time the outer ring was opened until after the inner ring was cut. We combined mean circumcision times for the first 30 and last 30 circumcisions at each of the 5 peripheral sites.

Wound healing was evaluated as the proportion of clients who were completely healed by the day 42 visit and was defined as no scab present, with complete re-epithelialization of the wound.

AEs were classified as in the WHO/Population Services International (PSI) Adverse Event Action Guide.^19^ Wound dehiscence, originally described in terms of number of disrupted sutures, needed a modified description/classification given that sutures are not present in ShangRing circumcision. Moderate wound dehiscence after ShangRing circumcision was defined as a mucocutaneous gap greater than 1 cm between the edges of the wound, along the shaft of the penis. We also considered the presence of a scab and healthy pink granulation tissue as part of the normal healing process after ShangRing circumcision, although it would appear otherwise after conventional circumcision.

To evaluate the comparative satisfaction of the participants circumcised in the 2 groups, we used the study completion interview responses to questions about how satisfied each participant had been with the appearance of his healed penis and whether he would recommend the ShangRing to a friend or family member.

### Statistical Analysis

The χ^2^ test was used to compare proportions of participants healed by day 42 and AE distributions (moderate and severe). The Student *t* test (paired) was used to compare the mean circumcision times of the first 30 and last 30 procedures at each of the peripheral sites. The significance level was set to 0.05.

### Ethical and Regulatory Review

Ethical committee approvals for the trial were obtained from FHI 360, the Kenya Medical Research Institute, and the University of Zambia Biomedical Research and Ethics Committee. Regulatory approvals were obtained from the Kenya Pharmacy and Poisons Board and the Zambian Pharmaceutical Regulatory Authority.

## RESULTS

A total of 6 experienced trainers and 10 newly trained MCPs were included in this analysis. Five (50%) of the newly trained providers were male. Five hundred twenty-one circumcisions were included in this analysis, 406 (77.9%) of which were performed by the newly trained ShangRing providers, whereas the remaining 115 (22.1%) were performed by the experienced providers at the main study site (Table 1).

**TABLE 1.**
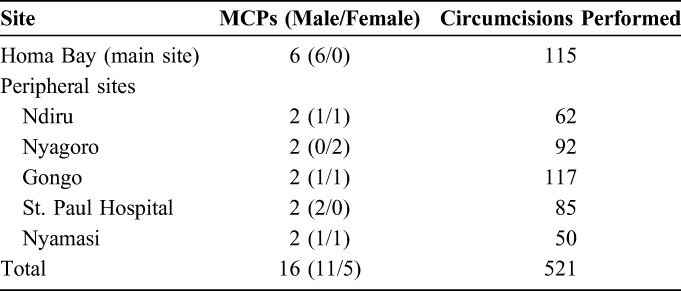
Demography, Distribution, and Circumcisions Per Site

The mean times required to perform the ShangRing circumcisions were 6.2 ± 1.6 and 6.2 ± 2.0 minutes by the newly trained and experienced groups, respectively (*P* = 0.45). Experienced MCPs took significantly less time (2.2 ± 1.4 minutes) to complete ShangRing removals compared with the newly trained ones (3.2 ± 1.5 minutes, *P* < 0.01). There was a significant decrease in time taken to complete the circumcision in 2 of the 5 sites where the newly trained MCPs were stationed (Table 2).

**TABLE 2.**
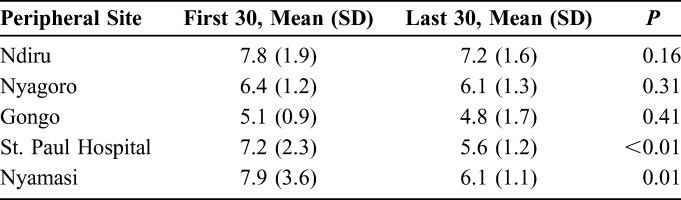
Time Taken to Complete the First 30 and Last 30 Circumcisions

Mean pain scores 30 minutes after the end of the circumcision were 2.5 ± 1.6 and 3.2 ± 1.6 for the newly trained and experienced MCPs, respectively (*P* = 0.65).

By the day 42 visit, 97.5% and 94.8% of men circumcised by the newly trained and the experienced teams, respectively, demonstrated complete wound healing (*P* = 0.13).

No serious AEs were observed. A total of 11 AEs were observed. No severe and 8 (2.0%) moderate AEs were seen among participants circumcised by the newly trained MCPs versus 1 (0.9%) severe and 2 (1.7%) moderate AEs among participants circumcised by the experienced group (*P* = 0.16). For the experienced providers, there was one severe AE, a severe wound dehiscence requiring surgical intervention in the course of healing, and 2 moderate AEs that were cases of moderate wound dehiscence. Similarly, 4 (50%) of the AEs observed from the newly trained MCPs were associated with moderate wound dehiscence. There were 2 further cases of infection which were managed with oral antibiotics: 1 case of moderate pain that interfered with normal activity and sleep and 1 case where insufficient skin had been removed. Apart from the single severe AE representing a wound dehiscence, all AEs in both groups resolved with conservative management with no long-term sequelae.

There was no difference (*P* = 0.23) in the percentage of men who were very satisfied with the appearance of the healed circumcision wound between participants circumcised by the newly trained (400/406, 98.5%) versus the experienced providers (111/115, 96.5%). Overall, 99.8% and 99.1% of those circumcised by the newly trained and experienced MCPs, respectively, said they would recommend the ShangRing to a friend.

## DISCUSSION

This analysis compared surgical outcomes for newly trained ShangRing MCPs versus experienced ones. Our findings show that clinicians with varied experience in conventional MC techniques are able to conduct safe and effective ShangRing circumcisions after a 1-week training course followed by 2 to 3 weeks of intermittent supportive supervision by more experienced trainers.

There was no significant difference in time taken to complete the ShangRing circumcisions between the 2 groups, although newly trained providers required one additional minute for ring removal compared with their experienced counterparts. A learning curve effect was seen in terms of reducing circumcision duration over time for the newly trained MCPs, which was significant in 2 of their sites. An analogous effect has been seen with conventional circumcision, where 40%–45% reductions in procedure time were noted after the first 100 procedures performed.^20,21^

Participants circumcised by newly trained MCPs did not report higher pain scores 30 minutes after the procedures compared with those circumcised by the experienced providers. There was also no significant difference in the rate of wound healing between the 2 groups.

There was no significant difference in the proportions of moderate and severe AEs between the groups. This differs from what has been seen in studies of conventional MC, where rates of AEs have been shown to reach as high as 8.8% but decrease significantly with provider experience after 100 procedures have been performed.^20–22^

In our study, the 10 newly trained providers had varying experience in conventional techniques but only needed to perform a minimum of 7 ShangRing circumcisions during training before being deemed competent. Achieving satisfactory skill competence in ShangRing may be easier to attain, even for an MCP who has been newly trained in conventional techniques but has not performed as many as 100 conventional procedures.

Study limitations included exclusion of the Zambian study population, and heterogeneity in the previous surgical experience of the newly trained providers in Kenya.

Our results demonstrate that the ShangRing circumcision technique is easy to learn and master and that newly trained providers can safely conduct ShangRing circumcisions in routine service settings. Use of the ShangRing reduces interprovider variability while producing consistent surgical outcomes even with new trainees. ShangRing circumcision appears to have a relatively short learning curve, with few AEs or other negative outcomes seen, even shortly after training. This study contributes to the growing body of evidence demonstrating that the ShangRing could facilitate rapid scale-up of VMMC services in sub-Saharan Africa.

## References

[1] Bailey RC Moses S Parker CB Male circumcision for HIV prevention in young men in Kisumu, Kenya: a randomised controlled trial. Lancet. 2007;369:643–656. 17321310 10.1016/S0140-6736(07)60312-2

[2] Gray RH Kigozi G Serwadda D Male circumcision for HIV prevention in men in Rakai, Uganda: a randomised trial. Lancet. 2007;369:657–666. 17321311 10.1016/S0140-6736(07)60313-4

[3] Auvert B Taljaard D Lagarde E Randomized, controlled intervention trial of male circumcision for reduction of HIV infection risk: the ANRS 1265 trial. PLoS Med. 2005;2:1112–1122. 10.1371/journal.pmed.0020298 PMC1262556 16231970

[4] Auvert B Taljaard D Rech D Association of the ANRS-12126 male circumcision project with HIV levels among men in a South African township: evaluation of effectiveness using cross-sectional surveys. PLoS Med. 2013;10:e1001509. 24019763 10.1371/journal.pmed.1001509 PMC3760784

[5] Mehta SD Moses S Agot K The long term efficacy of medical male circumcision against HIV acquisition. AIDS. 2013;27:2899–2907. 23835501 10.1097/01.aids.0000432444.30308.2d

[6] Gray R Kigozi G Kong X The effectiveness of male circumcision for HIV prevention and effects on risk behaviors in a posttrial follow-up study. AIDS. 2012;26:609–615. 22210632 10.1097/QAD.0b013e3283504a3f PMC4296667

[7] NASCOP. National Guidance for VMMC in Kenya—2008. 2008 Available at: http://malecircumcision.org/programs/documents/KenyaMCguidance.pdf. Accessed February 5, 2015.

[8] UNAIDS. New Data on Male Circumcision and HIV Prevention: Policy and Programme Implications. Available at: http://data.unaids.org/pub/Report/2007/mc_recommendations_en.pdf. Accessed February 5, 2015.

[9] UNAIDS, WHO. Joint Strategic Action Framework to Accelerate the Scale-up of Voluntary Medical Male Circumcision for HIV Prevention in Eastern and Southern Africa. 2011:36 Available at: http://whqlibdoc.who.int/unaids/2011/JC2251E_eng.pdf?ua=1. Accessed February 5, 2015.

[10] USAID. The Potential Cost and Impact of Expanding Male Circumcision in 14 African Countries. 2009 Available at: http://www.malecircumcision.org/programs/documents/14_country_summary11309.pdf. Accessed March 3, 2015.

[11] Sgaier SK Reed JB Thomas A Achieving the HIV prevention impact of voluntary medical male circumcision: lessons and challenges for managing programs. PLoS Med. 2014;11:e1001641. 24800840 10.1371/journal.pmed.1001641 PMC4011573

[12] Framework for Clinical Evaluation of Devices for Male Circumcision. Geneva: World Health Organization HIV/AIDS Programme 2012. Available at: http://apps.who.int/iris/bitstream/10665/75954/1/9789241504355_eng.pdf. Accessed December 5, 2014.

[13] Sokal DC Li PS Zulu R Field study of adult male circumcision using the ShangRing in routine clinical settings in Kenya & Zambia. J Acquir Immune Defic Syndr. 2014;67:430–437. 25162816 10.1097/QAI.0000000000000321

[14] Sokal DC Li PS Zulu R Randomized controlled trial of the Shang ring versus conventional surgical techniques for adult male circumcision: safety and acceptability. J Acquir Immune Defic Syndr. 2014;65:447–455. 24583615 10.1097/QAI.0000000000000061

[15] Kigozi G Musoke R Watya S The acceptability and safety of the Shang Ring for adult male circumcision in Rakai, Uganda. J Acquir Immune Defic Syndr. 2013;63:617–621. 23614991 10.1097/QAI.0b013e3182968dda PMC3805675

[16] Auvert B Taljaard D Lagarde E Randomized, Controlled Intervention Trial of Male Circumcision for Reduction of HIV Infection Risk: The ANRS 1265 Trial. PLoS Med. 2005;2:e298. 16231970 10.1371/journal.pmed.0020298 PMC1262556

[17] Frajzyngier V Odingo G Barone M Safety of adult medical male circumcision performed by non-physician clinicians in Kenya: a prospective cohort study. Glob Health Sci Pract. 2014;2:93–102. 25276565 10.9745/GHSP-D-13-00120 PMC4168600

[18] Masson P Li PS Barone MA The ShangRing device for simplified adult circumcision. Nat Rev Urol. 2010;7:638–642. 20938437 10.1038/nrurol.2010.167

[19] Rech D Dickson KE Samkange CA Adverse Event Action Guide for Voluntary Medical Male Circumcision by Surgery. 2011 Available at: http://www.malecircumcision.org/programs/documents/AE Guide Job Aid_April 14 2014_final version.pdf. Accessed February 25, 2015.

[20] Herman-Roloff A Bailey R Agot K Factors associated with the safety of voluntary medical male circumcision in Nyanza province, Kenya. Bull World Health Organ. 2012;90:773–781. 23109745 10.2471/BLT.12.106112 PMC3471059

[21] Kiggundu V Watya S Kigozi G The number of procedures required to achieve optimal competency with male circumcision: findings from a randomized trial in Rakai, Uganda. BJU Int. 2009;104:529–532. 19389002 10.1111/j.1464-410X.2009.08420.x PMC2748867

[22] Krieger JN Bailey RC Opeya JC Adult male circumcision outcomes: experience in a developing country setting. Urol Int. 2007;78:235–240. 17406133 10.1159/000099344

